# Comparing the Efficacy and Safety of Low-Carbohydrate Diets with Low-Fat Diets for Type 2 Diabetes Mellitus Patients: A Systematic Review and Meta-Analysis of Randomized Clinical Trials

**DOI:** 10.1155/2021/8521756

**Published:** 2021-12-06

**Authors:** Shunhua Li, Lu Ding, Xinhua Xiao

**Affiliations:** Department of Endocrinology, Key Laboratory of Endocrinology, Ministry of Health, Peking Union Medical College Hospital, Diabetes Research Center of Chinese Academy of Medical Sciences and Peking Union Medical College, Beijing 100730, China

## Abstract

**Introduction:**

To compare the efficacy of low-carbohydrate diets (LCDs) with low-fat diets (LFDs) in body weight and glycemic control for type 2 diabetes mellitus (T2DM) patients, and their cardiovascular and renal safety.

**Methods:**

We searched PubMed, Ovid, Embase databases, Cochrane Central Register of Controlled Trials (CENTRAL), and ClinicalTrials.gov from inception to April, 2021. Randomized controlled trials (RCTs) which lasted more than 3 months were included. The primary outcomes are the mean change from baseline in glycated haemoglobin (HbA1c) and body weight loss. Secondary outcomes included mean difference in lipid parameters, blood pressures, and serum creatinine.

**Results:**

Totally, 12 RCTs met inclusion criteria representing 761 patients. Compared with LFDs, treatment with LCDs achieved significant reduced HbA1c by 0.35% (95% CI: −0.45, −0.24; *P* < 0.00001). LCDs appeared to be more beneficial in decreasing body weight than LFDs (WMD = −2.99 kg; 95% CI: −4.36, −1.63; *P* < 0.0001), especially in the subgroup that used VLCDs (WMD = −9.49 kg; 95% CI: −12.88, −6.09, *P* < 0.00001). For cardiovascular risk factors, the LCD interventions significantly reduced TG concentration (WMD: −0.20 mmol/l; 95% CI: −0.31, −0.10; *P* = 0.0001) and increased HDL-C concentration (WMD: 0.09 mmol/l; 95% CI: 0.05,0.13; *P* < 0.00001). Subgroup analyses demonstrated that the difference in HbA1c, TG, and HDL-C between two dietary restrictions respectively lasted up to 1.5 and 2 years, whereas the beneficial effects of body weight loss diminished over time and disappeared after 2 years. LCDs were not associated with decreased level of TC or LDL-C, neither SBP nor DBP in comparison with LFDs. Moreover, no significant difference in serum creatinine could be found among such two diet interventions.

**Conclusions:**

LCDs are superior to LFDs for T2DM patients in improving HbA1c and reducing body weight, with a rewarding effect of some cardiovascular risk factors in a longer-term diabetes management. However, available data are insufficient to evaluate the association between diet interventions and renal safety. Future larger longer-term follow-up clinical trials are needed to provide more evidence about the sustainable effects and safety of LCDs compared with LFDs.

## 1. Introduction

Diabetes mellitus is a chronic and complex disease, which has posed unprecedented challenges to public health and economy. The International Diabetes Federation (IDF) recently announced that more than 463 million adults worldwide were living with diabetes in 2019. If this growing trend continues, the number of diabetes patients will increase to 700 million in 2045. Among them, type 2 diabetes mellitus (T2DM) accounts for the vast majority (approximately 90%) of global diabetes, becoming a “catastrophe of the twenty-first century” [1].

Current treatment guidelines for T2DM mainly focus on multiple drug therapies to lower blood glucose and the associated increased cardiovascular disease risks. However, the expectation of T2DM patients' life quality is still substantially reduced [2]. Lifestyle management, composed of nutrition therapy and physical activity, is the cornerstone of diabetes care. But behavior interventions alone may not be sufficient to maintain normal blood glucose for a long time due to the progression of T2DM. It is worth noting that nutritional therapy remains a crucial part after the initiation of medication and should be incorporated with the overall treatment procedure [3].

Structured dietary interventions are often recommended as an important component of lifestyle management for T2DM. It helps to achieve glycemic goals by lowering HbA1c [4]. Mediterranean style [5, 6], low-carbohydrate [7–9], and vegetarian or plant-based [10, 11] are examples of healthful eating patterns, which have been reported to generate positive effects. However, none of these diet interventions has been proven to be consistently superior [12–14].

The effects of low-fat diets (LFDs) and low-carbohydrate diets (LCDs) consistent in T2DM patients have been commonly compared. Reducing the total carbohydrate intake has been approved by the American Diabetes Association (ADA) as the strongest evidence for improving glycemia [15]. However, the most profitable quantity of carbohydrate intake for diabetics has not been determined. In addition, the higher fat and protein content in LCDs has raised concerns for their safety. Among adults with diabetes who already have a high risk of kidney disease or cardiovascular disease (CVD), the potential impacts of LCDs on renal insufficiency, ketoacidosis, and CVD risk may attenuate or eliminate the benefits of weight loss [16]. The traditional energy-reduced, carbohydrate restricted, low-protein LFD has sparked a similar debate, as there is evidence that suggests fat and protein independently suppress postprandial glucose responses elicited by dietary carbohydrate [17–19]. It is recommended that reducing the percentage of total calories from saturated fats may be more efficient than limiting the total amount of fat [15].

The incidence, prevalence, and costs of T2DM are rapidly increasing and usually are in parallel with obesity and cardiovascular diseases, embracing a dysfunctional metabolic network. There is strong and consistent evidence that moderate sustained weight loss not only benefits T2DM treatment, but also improves other CVD risk factors such as hypertension and dyslipidemia [15]. Taking the above variables into account, the purpose of our meta-analysis is to assess differences between LCDs and LFDs with respect to glycemic control, body weight, lipid profile, blood pressure, and renal safety in adults with T2DM.

## 2. Methods

This systematic review and meta-analysis was performed under the guidance of the Cochrane Handbook for Systematic Reviews of Interventions [20] and was reported according to the PRISMA Statement [21].

### 2.1. Search Strategy

We searched the following databases from inception to April, 2020, to identify eligible randomized controlled trials (RCTs) without restrictions on language: PubMed, Ovid, Embase databases, Cochrane Central Register of Controlled Trials (CENTRAL), and ClinicalTrials.gov. We researched reference lists of previously published relevant reviews and studies to make sure no papers were missed. Full details of our literature search are outlined in Supplementary Material Table S1.

### 2.2. Selection Criteria

Studies were included if they fulfilled the following criteria: (1) they were RCTs; (2) study populations were T2DM patients; (3) treatment interventions included LCD (<26% of daily calories from carbohydrates or <130 g/day) and LFD (as defined by authors); (4) they reported the mean change in glycated haemoglobin (HbA1c) and weight loss from baseline, and (5) duration of trial was at least 12 weeks.

Trials that enrolled participants with type 1 diabetes or pregnancy as part of the study population were excluded. Enteral feeds and additional weight-loss pharmacological intervention were also excluded. If more than one article was published on an overlapping population, the most comprehensive article was included in the meta-analysis.

Two investigators (Shunhua Li and Lu Ding) screened articles independently. Any discrepancies in the final inclusion eligibility were resolved by discussion with a third author (Xinhua Xiao).

### 2.3. Data Extraction

The study information of patients was recorded, including the first author, year of publication, study region, sample size, composition of LCD and LFD, duration of intervention, and the percentage of missing participant outcome data. Baseline participant characteristics included age, gender, anthropometrics, and duration of T2DM.

We extracted the outcomes of mean differences based on changes from baseline. The primary outcome was changed in glycated haemoglobin (HbA1c, %) and body weight lost (kg). Secondary outcomes of interest included total, low-density lipoprotein and high-density lipoprotein cholesterol (TC, HDL-C, LDL-C, mmol/l), triglycerides (TG, mmol/l), systolic and diastolic blood pressure (SBP, DBP, mmHg), and creatinine (mg/dL). When these research measures have outcomes in multiple ways, we would transform the results to a unified scale.

Data extraction was done independently by two authors (Shunhua Li and Lu Ding). Any disagreements were resolved and adjudicated by the third reviewer (Xinhua Xiao) when necessary.

### 2.4. Statistical Analysis

All statistical analyses were performed with Review Manager (RevMan, version 5.4) with a *P* < 0.05 considered statistically significant. The differences of continuous data were analyzed by mean differences (MDs) with 95% confidence intervals (CIs). All the standard errors of the mean (SEM) were transformed into the standard deviation (SD) by using the formula SD = SEM × $\sqrt{N}\;$ [20]. When the SD was not available, 95% CIs were used where possible. If studies only provided baseline and endpoint data, MDs from baseline and SD of the change were calculated by applying the formula of Follmann et al. [22] and assuming a correlation of 0.5 between the baseline and final measures within each group, as follows:

(1) $$\begin{array}{c} \sqrt{{\left({SE}_{\text{baseline}}\right)}^{2}+{\left({SE}_{\text{final}}\right)}^{2}-2\times 0.5\times {SE}_{\text{baseline}}\times \left({SE}_{\text{final}}\right)}. \end{array}$$

The statistical heterogeneity between the included studies was evaluated though the *Q* tests and *I*^2^ statistics. The fixed-effects model was selected as the primary analysis. *P* < 0.10 or *I*^2^> 50% was regarded as statistical heterogeneity; then, a random-effects model was used to secondarily repool data and a subgroup analysis was preformed to explore the potential source of heterogeneity.

### 2.5. Study Risk of Bias Assessment

We used version 2.0 of the Cochrane Risk-of-Bias (RoB 2.0) instrument for RCTs and assessed each of the RoB domains as “high,” “low,” or “some concern” using the Excel file provided by the RoB 2.0 development team [23]. To assess for the possibility of publication bias, we visually inspected funnel plots for primary outcomes. Furthermore, Egger linear regression test was performed by using STATA version 12.0 (StataCorp LP, College Station, Texas, USA).

## 3. Results

### 3.1. Trial Characteristics

**Figure 1:**
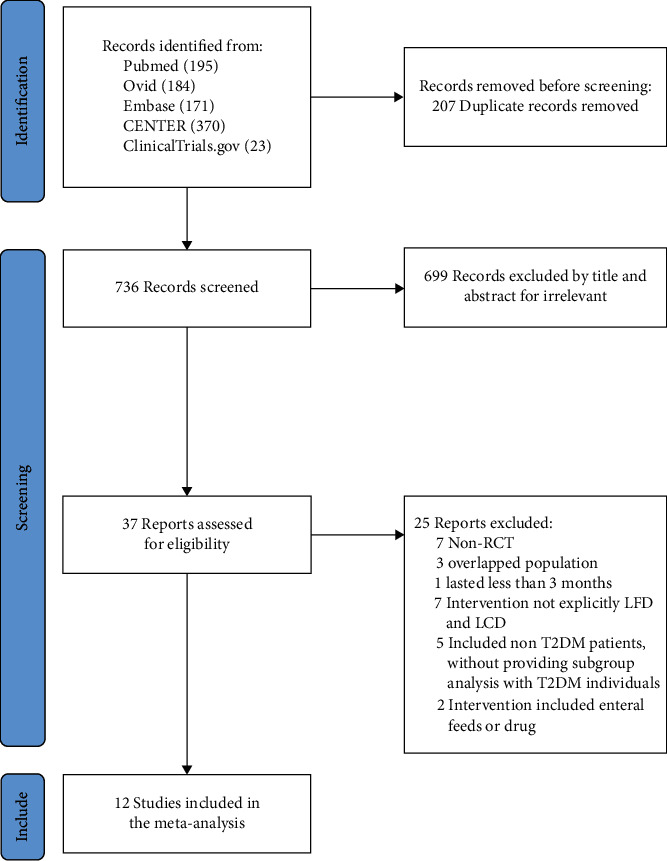
Flow diagram of study selection.

Our search yielded 943 citations, of which 12 RCTs enrolling 761 T2DM patients met the inclusion criteria in this meta-analysis (Figure 1). The baseline characteristics of the 12 trials were listed in Table 1.

Studies included in this review had intervention periods varying from 3 months to 2 years and participants' numbers ranging from 17 to 115. To sum up, the mean age of participants of each intervention arm ranged from 53 to 65 years, the proportion of women ranged from 23% to 89%, and the mean baseline HbA1c ranged from 5.9% to 9.1%. Trails used various carbohydrate restriction thresholds: 9 articles [24–35] used LCD (<130 g/day or 26% total energy), whereas another 3 articles [27, 29, 30] used very-low-carbohydrate diet (VLCD, 20–50 g/day or <10% of the 2000 kcal/day diet).

All 12 researches provided the complete data on HbA1c and loss of body weight. 11 studies [24–35] provided the data of TG levels. 10 studies [24–35] investigated the outcome of LDL-C and HDL-C, and 6 studies [24, 26, 28, 32, 34, 35] compared the effect of LCD and LFD on TC concentration. 6 studies [25–32, 35] reported SBP and 5 studies [27–32, 35] presented DBP. Moreover, only 4 studies [26, 31, 32, 34] covered serum creatinine at endpoint.

**Figure 2:**
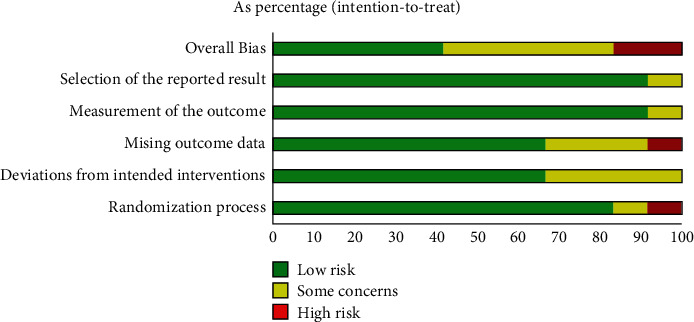
Risk of bias by outcome (percentage).

Risk of bias is summarized in Figure 2. Overall, 60% of outcomes were rated as having some concern or high risk of bias, and 40% of outcomes were rated as having low risk of bias. The missing outcome data and deviations from intended interventions were the poorest-reported domain of bias risk, with only 66.7% of trials having “low risk.” Due to the nature of the intervention, dropouts were common in the included studies. 8 of 12 trails reported missing participant outcome data, with 2 reporting more than 20% of data being missing.

### 3.2. Glycemic Control

**Figure 3:**
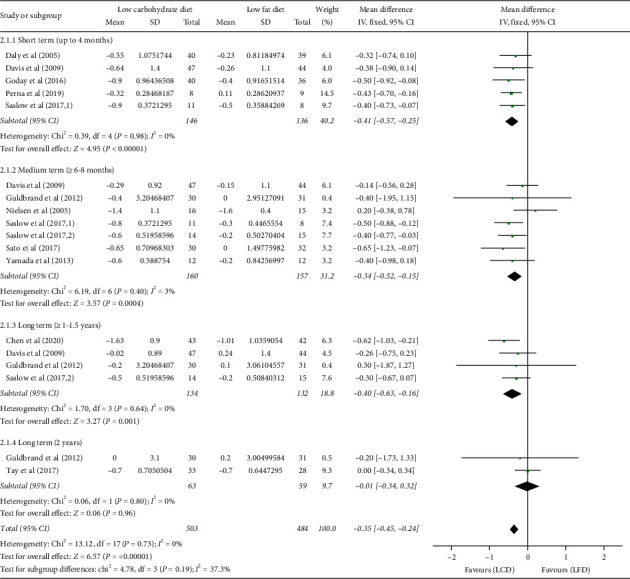
Forest plot for the effect of restriction diets on HbA1c. LCD: low-carbohydrate diet; LFD: low-fat diet; HbA1c: glycated haemoglobin; CI: confidence interval; SD: standard deviation.

This outcome was assessed on 5, 7, 4, and 2 trials at 3–4, 6–8, 12–18, and 24 months, respectively (Figure 3). The overall pooled result demonstrated that LCDs significantly reduced HbA1c by 0.35% (95% CI: −0.45, −0.24; *P* < 0.00001) compared with LFDs. Subgroup analyses according to length of follow-up revealed the beneficial effect of LCDs on HbA1c started from short terms (3–4 months: WMD = −0.41%; 95% CI: −0.57, −0.25, *P* < 0.00001; 6–8 months: WMD = −0.34%; 95% CI: −0.52, −0.15, *P* = 0.0004), which persisted at 1.5 years (WMD = −0.4%; 95% CI: −0.63, −0.16, *P* = 0.001) and disappeared at 2 years (WMD = −0.01%; 95% CI: −0.34, −0.32, *P* = 0.96). We used the fixed-effects model for analysis because no significant heterogeneity was found in the test (I^2^ = 0%, *P* = 0.73).

### 3.3. Body Weight Loss

**Figure 4:**
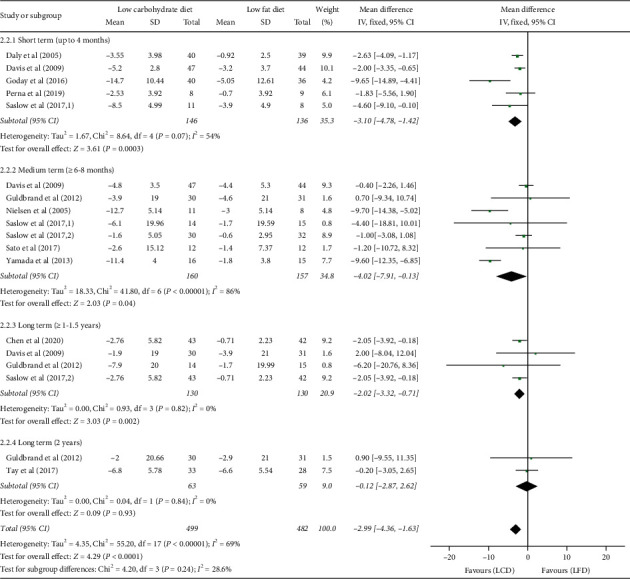
Forest plot for the effect of restriction diets on body weight loss. LCD: low-carbohydrate diet; LFD: low-fat diet; CI: confidence interval; SD: standard deviation.

This outcome data were analyzed with a random-effects model due to a moderate heterogeneity (*I*^2^ = 69%, *P* < 0.00001), which is shown in Figure 4. Overall, LCDs appeared to be more beneficial in decreasing body weight than LFDs (WMD = −2.99 kg; 95% CI: −4.36, −1.63; *P* < 0.0001). It is worth noting that LCDs significantly decreased body weight in the subgroup which lasted less than 8 months (3–4 months: WMD = −3.10 kg; 95% CI: −4.78, −1.42, *P* = 0.0003; 6–8 months: WMD = −4.02 kg; 95% CI: −7.91, −0.13, *P* = 0.04), but the difference diminished over time (1–1.5 years: WMD = −2.02 kg; 95% CI: −3.32, −0.71, *P* = 0.002), and even no significant effect was observed in 2-year follow-up subgroup (WMD = −0.12 kg; 95% CI: −2.87, 2.62, *P* = 0.93).

Stratified analysis was conducted for the different proportion of carbohydrate in carbohydrate-restricted diets to identify possible sources of heterogeneity (Supplementary Material Figure S1). Among subgroups of patients treated with LCDs compared with patients treated with LFDs, body weight had a slight drop (WMD = −1.57 kg; 95% CI: −2.41, −0.73, *P* = 0.0003). Interestingly, the reduction in body weight tended to be greater in the subgroup that used VLCD (WMD = −9.49 kg; 95% CI: −12.88, -6.09, *P* < 0.00001).

### 3.4. Cardiovascular Risk Factors

**Figure 5:**
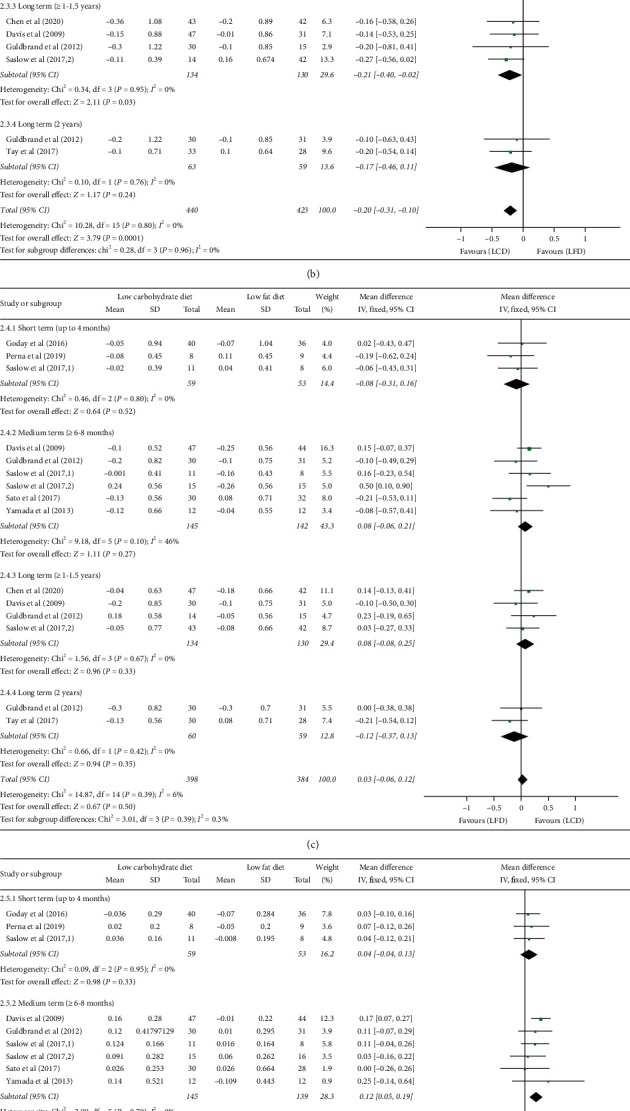
Forest plot for the effect of restriction diets on TC (a), TG (b), LDL-C (c), HDL-C (d), SBP (e), and DBP (f). TC: total cholesterol; HDL-C: high-density lipoprotein cholesterol; LDL-C: low-density lipoprotein cholesterol; TG: triglycerides; LCD: low-carbohydrate diet; LFD: low-fat diet; CI: confidence interval; SD: standard deviation.

The pooled results of blood lipid concentrations are shown in Figure 5. In general, there was a significant decrease in the concentration of TG (WMD: −0.20 mmol/l; 95% CI: -0.31, -0.10; *P* = 0.0001) and a significant increase in the level of HDL-C (WMD: 0.09 mmol/l; 95% CI: 0.05,0.13; *P* < 0.00001) in subjects who consumed LCDs compared with LFDs. However, there was no significant difference in the reductions of TC (WMD: 0.11 mmol/l; 95% CI: −0.03, 0.25; *P* = 0.12) and LDL-C (WMD: 0.03 mmol/l, 95% CI: −0.06, 0.12; *P* = 0.50) at any time point within the comparison of LCDs and LFDs. Meanwhile, meta-analysis on those four outcomes indicated that no statistical heterogeneity was found (TC : *I*^2^ = 0%, *P* = 0.68; TG : I^2^ = 0%, *P* = 0.80; LDL-C : I^2^ = 6%, *P* = 0.39; HDL-C : *I*^2^ = 0%, *P* = 0.94), and thus fixed-effects model was used.

Subgroup analyses in different study duration indicated that LCDs induced a greater increase in the level of HDL-C at almost all time points (6–8 months: WMD = 0.12 mmol/l; 95% CI: 0.05, 0.19, *P* = 0.0004; 1–1.5 years: WMD = 0.07 mmol/l; 95% CI: 0.01, 0.13, *P* = 0.03; 2 year: WMD = 0.12 mmol/l; 95% CI: 0.04, 0.20, *P* = 0.003), except at the shortest time window (3–4 months), where no difference in effects could be found between the two diets (WMD: 0.04 mmol/l; 95% CI: -0.04, 0.13; *P* = 0.33). With regard to the change from baseline in the concentration of TG, there was a trend in favour of LCDs in the first three time windows (3–4 months: -0.25 mmol/l; 95% CI: -0.47, -0.03; *P* = 0.03; 6–8 months: WMD = -0.18 mmol/l; 95% CI: -0.36, -0.00, *P* = 0.05; 1–1.5 years: WMD = −0.21 mmol/l; 95% CI: −0.40, −0.02, *P* = 0.03); only the data reported beyond 2 years showed unclear clinical importance of LCDs on TG level (WMD: −0.17 mmol/l; 95% CI: −0.46, 0.11; *P* = 0.24).

No difference was found in SBP (WMD: 0.83 mmHg; 95% CI: −2.01, 3.67; *P* = 0.57) or DBP (WMD: 0.23 mmHg; 95% CI: −1.71, 2.17; *P* = 0.82), without significant heterogeneity (SBP : *I*^2^ = 0%, *P* = 0.61; DBP : I^2^ = 0%, *P* = 0.64).

**Figure 6:**
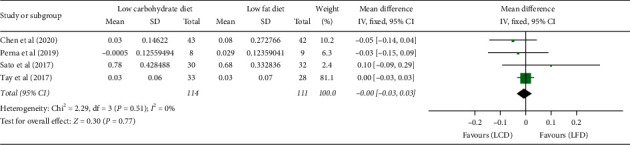
Forest plot for the effect of restriction diets on serum creatinine. LCD: low-carbohydrate diet; LFD: low-fat diet; CI: confidence interval; SD: standard deviation.

LCD compared with LFD resulted in no significant difference in serum creatinine (WMD: 0 mg/dl, 95% CI: −0.03, 0.03; *P* = 0.77), without heterogeneity (I2 = 0%, *P* = 0.51) among the studies (Figure 6).

### 3.5. Publication Bias

Visual inspection of funnel plots (Supplementary Material Figure S2) and Egger test suggests no evidence of publication bias on primary outcomes, including HbA1c (*P* = 0.164) and body weight loss (*P* = 0.107).

## 4. Discussion

To the best of our knowledge, this meta-analysis is the first one to compare the efficacy and safety of LCDs and LFDs on glucose control, body weight loss, cardiovascular risk, and renal safety for type 2 diabetes management. We found that treatment with LCDs achieved more effective improvements in glycemic and body weight control, as well as the concentrations of TG and HDL-C. Since available data suggested that the duration of observation is a crucial point in the definition of efficacy [7, 36, 37], subgroup analyses based on different trail durations ranged from 3 months to 2 years were performed. Our study proved that a more favourable effect of LCDs on HbA1c and TG levels could persist up to 1.5 years. The result of HDL-C concentration suggested a longer-term effectiveness of LCDs, which lasted for 2 years. In terms of body weight loss, carbohydrate restriction more clearly improved this outcome in the short and medium terms up to 6–8 months, but the difference attenuated in 1–1.5 years and disappeared after 2 years. However, there is no significant superiority of LCDs in improving TC and LDL-C levels, neither SBP nor DBP. In contrast to LFDs, LCDs had no statistical difference in serum creatinine.

In this study, results showed that LCDs significantly reduced HbA1c compared with LFDs. The improvement in glucose metabolism and the alleviation of insulin sensitivity might be involved in the possible explanation for this positive finding. LCD might directly affect the output of hepatic glucose and the utilization of glucose through ketone bodies production [38, 39]. It may come as a surprise that our data demonstrated a more lasting effect of LCDs on glycemic control than other systematic reviews focusing on the effects of low-carbohydrate diets on metabolic outcome variables, which reported HbA1c rebounds after 6 or 12 months intervention [7, 37]. As opposed to our results, another meta-analysis showed that LCDs were not more effective than balanced diets and were even possibly harmful in the longer-term [36]. In fact, the HbA1c test is limited because it is an indirect measurement of average blood glucose. There are other indicators such as glycemic variability and postprandial and fasting glucose which need to be considered in the evaluation of T2DM remission. Moreover, the clinically important minimal difference for HbA1c has not been determined, and our inference with regard to clinical meaning is arguable. Therefore, we could not draw a conclusion arbitrarily although LCDs showed superior efficacy in reducing HbA1c; more comprehensive monitoring outcomes need to be concurrently assessed in the following exploration.

Results from a comprehensive meta-analysis of RCTs followed up for at least 12 months indicated LFDs do not make subject slimmer compared to higher fat dietary approaches of similar intensity [40]; this was supported by our results. Carbohydrate-restriction diets, in comparison with fat-restriction diets, identify a significant reduction of body weight. Moreover, the effect on weight loss was caused by the ratio of energy from carbohydrate to total energy because this merit was likely to be greater in diets with VLC content. In overweight and obese individuals with diabetes, modest weight loss was thought to be effective in improving insulin resistance [41], thereby extending the advantages of LCD for T2DM management. However, our data suggested that the greater weight loss led by LCDs disappeared more earlier than other improved metabolic variables. We also observed a tendency that body weight is regained to baseline value after medium term (about 6–8 months) in both diet arms, which could be attributed to the difficulties of adherence to dietary changes over relatively longer follow-up period. Besides, some preceding meta-analyses such as Nuttall et al. [42] illustrated that LCD improved HbA1c level without the loss of body weight compared with other dietary interventions. Therefore, further studies are necessary to identify whether the weight loss is the necessary requirement for the usefulness of LCDs or not.

Atherosclerotic cardiovascular disease (ASCVD) is the main cause of morbidity and mortality in diabetic patients. Hypertension and dyslipidemia, two common conditions coexisting with T2DM, are clear risk factors for both ASCVD and microvascular complications [15]. An underlying cause for concern of LCDs regarding cardiovascular safety is their increased fat intake, which could have adverse influences on lipid profile and other risk factors [43]. Notably, LCD not only did not worsen SBP, DBP, TC, or LDL-C concentrations of T2DM patients, but also even had more favourable effects on TG and HDL-C levels than LFD. A recent study identified a 6-week LCD intervention adding to the positive effect of weight loss in T2DM patients by inducing greater improvements in lipid profile [44], which was consistent with our results. However, the available data of 2-year time window in this meta-analysis were only from 2 of the trails [28, 32], which was insufficient for answering a clearly defined clinical question on the long-term benefit of these two dietary strategies for T2DM management. Moreover, although the evidence for total fat intake is disputable, there is a mushrooming number of research supporting the value of fat quality [40] for the prevention of CVD. Another meta-analysis investigated that the major increase in dietary fats of LCDs came from unsaturated fats, especially monounsaturated fats [41], which was subsequently confirmed to improve metabolic risk factors among T2DM patients [45]. According to the results of this meta-analysis, there was no evidence that LCD had any adverse effect on CVD, but stronger evidence still requires long-term trials with large sample sizes.

Observational data revealed that dietary protein intake is related to the progression of renal disease in patients with DM [46]. Considering that limiting proportion of carbohydrate is related to increased protein uptake, which has provoked the controversy on its renal safety, almost included trails [24–27, 31–34] listed impaired renal function or individuals already suffered from renal disease as exclusion criteria. However, only 4 studies [2, 31, 32, 34] provided available quantitative data on renal function at the end of follow-up. In such a small study population, no difference between the two restriction diet arms was detectable. Previous meta-analysis demonstrated the similar result [36]. Due to the possibility of publication bias, the data source from patients with normal renal function, and the limited number of included trials, this result should be considered cautiously.

Several limitations should be taken into account when interpreting the results of this meta-analysis. Due to practical constraints, the majority of trials were relatively small, limiting the precision of estimates of treatment effect. In addition, most studies considering the role of LCD and LFD in T2DM management did not consider for medication changes, quality of patients' life, and treatment satisfaction and thus cannot provide a comprehensive assessment of the efficacy of dietary interventions. Another potential limitation was that analyses were based on the randomized design original anticipated, rather than adhering to the actual dietary approach and/or macronutrient composition and caloric intake consumed. This meant that although patients were randomly assigned to various dietary groups, the details of their practical compliance with the diet prescription were beyond the scope of analyses. Notably, comparisons of the foods consumed for the different dietary interventions in each study were generally unavailable, the reader should bear in mind that the systematic errors in reporting energy intake may be induced by the low reproducibility of results from food intake surveys. Another major confounding factor was the independent influence of weight change on the other measured outcomes. It is difficult to distinguish the effect of weight loss on glycemic control and lipid profile, possibly interfering with difference between groups.

Considering several questions in the application of diet interventions remains unanswered at present, we believe that there is an abundant room for further progress in determining the efficacy and safety issues of LCDs and LFDs for T2DM treatment. The meta-analysis of Bravata et al. [47] depicted that the body weight change was connected with the limitation of caloric intake but not restricted carbohydrate content. Further work is required to establish the relationship between energy intake alteration and weight loss independent of the amounts of dietary carbohydrate and fat. The isocaloric prescription of diets is therefore recommended, which enables comparisons of the efficacy and positive health effects between dietary therapies in a long term, without the confounding effect of distinctions in total caloric intake and weight loss. Secondly, an indispensable issue for body weight control is the prolonged adherence of restriction dietary. Changes in the composition of macronutrient in a long-term trail possibly result in insulin metabolism adaptation to some extent. Larger size of trial with longer duration of intervention is required to better understand the persistence of the effects on glycemic and weight control in a broader population, and to track whether the metabolic health effects are sustained over time. Thirdly, high compliance depends on the intensity of provided intervention, with advanced professional support and subsidized food supplies, limiting universality of widespread community adoption. It cannot be firmly convinced that if more efforts are made to achieve consistent compliance with reduced energy intake, different consequences might happen. Future explores should be undertaken to incorporate these research findings into cost-effective community-based delivery models.

## 5. Conclusions

This systematic review and meta-analysis provided evidence that modifying the amounts of macronutrients can improve glycemic control, weight, and lipids in people with T2DM and especially highlighted that LCDs are superior to LFDs in improving HbA1c levels and controlling body weight, which was influenced by the carbohydrate content in the LCDs. The data of decreased TG and increased HDL-C concentrations supported LCDs might be more beneficial to cardiovascular risk factors than LFDs. Instead of being transient, these promoted metabolic outcomes persisted up to more than 1 year, indicating that the advantage effect of LCDs could exist in a relatively longer-term in T2DM management to a certain extent. However, there was no evidence to show that LCDs was effective in reducing TC and LDL-C levels, neither SBP nor DBP. Available data are insufficient to evaluate the association between the two diet approaches and renal safety.

To sum up, there is not a “one-size-fits-all” meal program for individuals with diabetes. Moving forward, the demand of further investigation is necessary to determine and validate those eating patterns that are optimal with respect to long-term efficacy, safety, and patient acceptability, while actively monitoring and adjusting diabetes medication as needed.

## Figures and Tables

**Table 1:** Characteristics of the trials and baseline of the enrolled participants.

Trails	Country	Number of participants(n) (LC/LF)	Attrition (%) (LC/LF)	Duration of diabetes (years)	Intervention measures		Age (years) (LC/LF)	Female (%) (LC/LF)
					LC group	LF group		
Chen et al. (2020) [24]	China	47/45	6/8.5	10.1/9.7	≤90 g of carbohydrate daily	≤30% fat	63.1/64.1	26/26
Daly et al. (2005) [25]	UK	51/51	21.5/23	NA	≤70 g of carbohydrate daily	Unclear	58.2/59.1	51/53
Davis et al. (2009) [26]	USA	55/50	14.5/12	NA	0–25 g increasing by 5 g increments each week	25% fat	54/53	45/37
Goday et al. (2016) [27]	Spain	45/44	11/18	NA	50 g of carbohydrate daily	<30% fat	54.89/54.17	67/64
Guldbrand et al. (2012) [28]	Sweden	30/31	0/0	NA	20% carbohydrate	30% fat	61.2/62.7	53/58
Saslow et al. (2017,1) [29]	USA	12/13	8/46	5.3/5.7	20–50 g of carbohydrate daily	Unclear	53/58.2	50/69
Saslow et al. (2017,2) [30]	USA	16/18	12.5/16.7	NA	20–50 g of carbohydrate daily	45–50% carbohydrate	64.8/55.1	56/89
Sato et al. (2017) [31]	Japan	33/33	9/3	14/13	130g of carbohydrate daily	50–60% carbohydrate	60.5/58.4	23/25
Tay et al. (2017) [32]	Australia	58/57	43/51	6/8	14% carbohydrate	<30% fat	58/58	36/49
Yamada et al. (2013) [33]	Japan	12/12	0/0	8.9/9.5	70–130 g of carbohydrate daily	≤25% fat	63.3/63.2	42/58
Perna et al. (2019) [34]	Italy	8/9	0/0	NA	<125 g of carbohydrate daily	25–30% fat	59.50/67.78	35.9/29.4
Nielsen et al. (2005) [35]	Sweden	16/15	0/0	13/8.5	20% carbohydrate	25% fat	57.1/58.6	NA

Trails	HbA1c (%) (LC/LF)	Bodyweight (kg) (LC/LF)	BMI (kg/m^2^)	TC (mg/dL) (LC/LF)	HDL-C (mg/dL) (LC/LF)	LDL-C (mg/dL) (LC/LF)	TG (mg/dL) (LC/LF)	Length of follow-up

Chen et al. (2020) [24]	8.47/8.7	69.69/68.34	27.31/26.55	180.12/174.95	47.21/43.61	103.02/103.87	163.70/177.81	1.5 years
Daly et al. (2005) [25]	9/9.11	101.6/102.3	35.4/36.7	188/191	46/47	NA	220/228	3 months
Davis et al. (2009) [26]	7.5/7.4	93.6/101	35/37	170/166	50/46	97/93	124/124	1 year
Goday et al. (2016) [27]	6.89/6.88	91.47/81.54	33.25/32.88	200.1/199.4	55.9/55.1	112.7/109.8	150.5/176.1	4 months
Guldbrand et al. (2012) [28]	7.5/7.2	91.4/98.8	31.6/33.8	174/166	44/42	104/93	151/159	2 years
Saslow et al. (2017,1) [29]	7.1/7.2	109.7/90.9	NA	174.1/151.5	45.7/53.9	96.9/90.5	NA	8 months
Saslow et al. (2017,2) [30]	6.6/6.9	99.9/97.5	35.9/36.9	106.5/160.3	48.4/45.8	88.7/98.1	102.6/158.9	1 year
Sato et al. (2017) [31]	8.0/8.3	74/73.6	26.7/26.5	NA	43.5/47.0	101.5/97	143.5/148.5	6 months
Tay et al. (2017) [32]	NA	101.7/101.6	34.2/35.1	174/166	46/50	97/92	142/124	2 years
Yamada et al. (2013) [33]	7.6/7.7	67/68.1	24.5/27	NA	62.8/59.8	99.8/112.2	141.7/155.2	6 months
Perna et al. (2019) [34]	5.90/5.99	81.56/88.52	30.30/32.41	183.75/174.67	48.50/46.44	103.23/97.42	159.38/154.00	3 months
Nielsen et al. (2005) [35]	NA	100.6/101.5	NA	NA	NA	NA	NA	6 months

LC: low-carbohydrate diet; LF: low-fat diet. HbA1c: glycated haemoglobin; BMI: body mass index; TC: total cholesterol; HDL-C: high-density lipoprotein cholesterol; LDL-C: low-density lipoprotein cholesterol; TG: triglycerides.

## Data Availability

The data used to support the findings of this study are available from the corresponding author upon request.

## Supplementary Materials

Supplementary Materials Figure S1: subgroup analyses of the effect of low-carbohydrate diet on weight loss indifferent proportion of carbohydrate. LCD: low-carbohydrate diet; LFD: low-fat diet; HbA1c: glycated haemoglobin; CI: confidence interval; SD: standard deviation. Figure S2: funnel plot of HbA1c (A) and weight loss (B). SMD: standardized mean difference; se (SMD): standard error of SMD. Table S1: search strategy.
