# Supplementary material for: Comparing the Efficacy and Safety of Low-Carbohydrate Diets with Low-Fat Diets for Type 2 Diabetes Mellitus Patients: A Systematic Review and Meta-Analysis of Randomized Clinical Trials
Source: Int J Endocrinol. 2021 Dec 6;2021:8521756. doi: 10.1155/2021/8521756 (PMC8668312; doi:10.1155/2021/8521756)
Supplement: Supplementary Materials — Figure S1: subgroup analyses of the effect of low-carbohydrate diet on weight loss indifferent proportion of carbohydrate. LCD: low-carbohydrate diet; LFD: low-fat diet; HbA1c: glycated haemoglobin; CI: confidence interval; SD: standard deviation. Figure S2: funnel plot of HbA1c (A) and weight loss (B). SMD: standardized mean difference; se (SMD): standard error of SMD. Table S1: search strategy. [file 8521756.f1.docx]

**Supplementary materials**

**
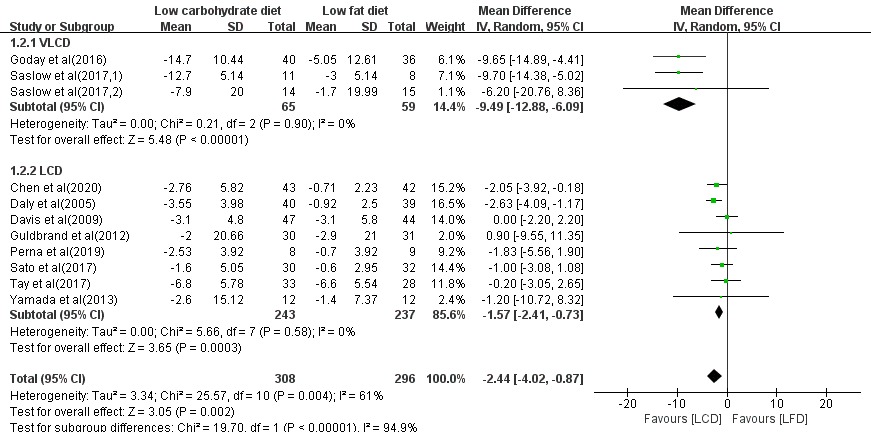
**

Figure S1. Subgroup analyses of the effect of low carbohydrate diet on weight loss indifferent proportion of carbohydrate.LCD: low-carbohydrate diet; LFD: low-fat diet; HbA1c: glycated haemoglobin; CI: confidence interval; SD: standard deviation.


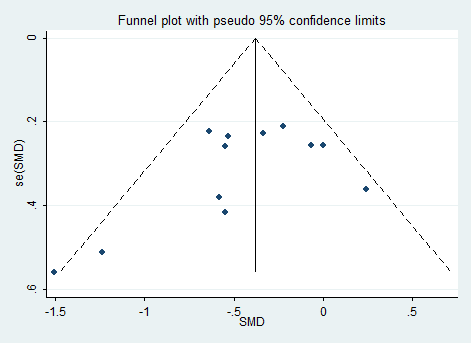


Figure S2. Funnel plot of HbA1c (A) and weight loss (B). SMD: standardized mean difference; se (SMD): standard error of SMD

**B**

**A**


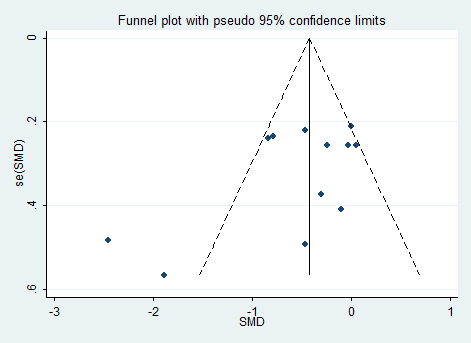


Table S1. Search Strategy

| **Data source** | **Search terms** |
| --- | --- |
| **PubMed** | (("diabete"[All Fields] OR "diabetes mellitus"[MeSH Terms] OR ("diabetes"[All Fields] AND "mellitus"[All Fields]) OR "diabetes mellitus"[All Fields] OR "diabetes"[All Fields] OR "diabetes insipidus"[MeSH Terms] OR ("diabetes"[All Fields] AND "insipidus"[All Fields]) OR "diabetes insipidus"[All Fields] OR "diabetic"[All Fields] OR "diabetics"[All Fields] OR "diabets"[All Fields]) AND ("low carbohydrate"[All Fields] OR "carbohydrate restricted"[All Fields] OR "ketogenic"[All Fields] OR "atkins"[All Fields]) AND ("diet"[MeSH Terms] OR "diet"[All Fields])) AND (randomized controlled trial[Filter]) |
| **Ovid** | #1 "randomized controlled trial".pt.  #2 (random$ or placebo$ or single blind$ or double blind$ or triple blind$).ti,ab.  #3 (retraction of publication or retracted publication).pt.  #4 or/#1-#3  #5 (animals not humans).sh.  #6 ((comment or editorial or meta-analysis or practice-guideline or review or letter or journal correspondence) not "randomized controlled trial").pt.  #7 (random sampl$ or random digit$ or random effect$ or random survey or random regression).ti,ab. not "randomized controlled trial".pt.  #8 #4 not (#5 or #6 or #7)  #9 (diabetes mellitus or type 2 diabetes mellitus or T2DM or diabet$).ab,ti,tw.  #10 (low carbohydrate OR carbohydrate restricted OR ketogenic OR atkins).ab,ti,tw.  #11 (diet).ab,ti,tw.  #12 #8 and #9 and #10 and #11 |
| **Embase** | ('diabetes mellitus'/exp OR 'diabetes' OR 'diabetes mellitus' OR 'diabetic') AND ('low carbohydrate' OR 'ketogenic diet'/exp OR 'diet, ketogenic' OR 'keto diet' OR 'ketogenic diet' OR 'ketogenous diet' OR 'ketotic diet' OR 'atkins diet'/exp OR 'atkin diet' OR 'atkin`s diet' OR 'atkins diet') AND ('randomized controlled trial (topic)'/exp |
| **CENTRAL** | Carbohydrate restricted diet OR low carbohydrate diet OR ketogenic diet OR Atkins diet O) AND (diabetes) in All Text - in Trials (Word variations have been searched) |
| **ClinicalTrials.gov** | carbohydrate-restricted diet OR low carbohydrate diet OR dietary carbohydrates OR ketogenic diet OR Atkins diet \| Studies With Results \| Interventional Studies \| Type2 Diabetes \| Adult |
